# Psychological characteristics of suicide attempters among undergraduate college students in China: a cross-sectional study

**DOI:** 10.1186/s12889-021-10370-2

**Published:** 2021-02-09

**Authors:** Bob Lew, Augustine Osman, Caryn Mei Hsien Chan, Won Sun Chen, Norhayati Ibrahim, Cun-Xian Jia, Ching Sin Siau

**Affiliations:** 1 Department of Social Psychology, Faculty of Human Ecology, Putra University of Malaysia, Serdang, Selangor Malaysia; 2 Department of Psychology, University of Texas at San Antonio, San Antonio, Texas USA; 3 Centre for Community Health Studies (ReaCH), Faculty of Health Sciences, Universiti Kebangsaan Malaysia, Kuala Lumpur, Malaysia; 4 Faculty of Health, Arts and Design, Swinburne University of Technology, Melbourne, Victoria, Australia; 5 Faculty of Health Sciences, Universiti Kebangsaan Malaysia, Kuala Lumpur, Malaysia; 6 School of Public Health, Cheeloo College of Medicine, Shandong University, Jinan, China; 7 Shandong University Center for Suicide Prevention Research, Jinan, China; 8 Centre for Community Health Studies (ReaCH),Faculty of Health Sciences, Universiti Kebangsaan Malaysia, Kuala Lumpur, Malaysia

**Keywords:** Suicide, Psychological stress, Depression, Undergraduate student, SBQ-R, China

## Abstract

**Background:**

There is a need to understand the psychological characteristics of suicide attempters to prevent future suicide attempts. This study aims to examine potential differences between individuals who have attempted suicide and those who have not done so, on several risk and protective measures.

**Method:**

Participants were 11,806 undergraduate students from seven provinces in China, of which 237 reported a non-fatal suicide attempt. We used the random numbers generator function within the SPSS to randomly select a control subset of 1185 participants to be used as the comparison group based on a 1:5 case-control ratio. Scores on three commonly used risk measures (depression, hopelessness, and psychache) and three protective measures (social support, self-esteem, and purpose in life) for suicidality were adopted to compare the responses of the two groups.

**Results:**

Suicide attempters had indicated higher Median scores for all three risk factor measurements. Suicide attempters also reported significantly lower Median scores for all three protective factor measurements compared to non-suicide attempters. The results suggest that the suicide attempters’ group had higher risks of suicidality compared to the non-attempter group.

**Conclusions:**

Suicide attempters continued to report higher scores of risk factors and lower scores of protective factors, indicating that they may continue to be at a higher likelihood of a suicide attempt. Key protective factors should be identified for each individual in order to deliver appropriate clinical interventions to reduce their risk of reattempting.

## Background

Suicide has grown to be an important public health concern with 800,000 deaths a year [1]. The World Health Organization estimates that there are approximately 25 cases of attempted suicide for every suicide death [1]. This modest estimate of 25 attempted cases for every suicide death gives us an estimation of 20 million people attempting suicide a year.

In China, the estimated suicide rate for year 2015 was about 8/100,000 population [2]. Based on an estimated population of 1.4 billion, this equates to at least 112,000 people a year. For every suicide death, approximately 135 other individuals experience substantive negative impact [3]. This works out to approximately 15 million people affected negatively by suicide in China each year.

Understanding and preventing suicide attempters from making future attempts is important [4]. Past suicide attempts have been documented as one of the highest risk factors for another suicide attempt [5], with up to 20% of attempters reattempting after their index attempt [6]. There is also a need to better understand risk factors and protective factors associated with suicide attempts. Risk factors are events or situations that are positively associated with suicidal behavior [7–9]. A meta-analysis by Ribeiro and colleagues [8] had indicated that individuals with hopelessness and depression had a 1.63 (95% CI 1.55–1.72) weighted mean odds ratio of attempting suicide. Psychache has also been found to demonstrate diagnostic accuracy for the indication of previous and recent suicide attempts among undergraduates [9]. Protective factors are significant for minimizing the desire to engage in suicide-related behaviors [10, 11]. In a meta-analysis of longitudinal studies by Soto-Sanz et al., youth with lower self-esteem were up to two times more like attempt suicide in the future [10]. Adolescents who reported lower social support from their parents, peer and school had greater odds or having a suicide attempt history [11]. Rodríguez and colleagues found that non-attempters had significantly higher purpose in life compared to self-harm and suicide attempt patients [12].

Amidst the declining trend of suicide rates in China in the past 20 years [13], there is a reported increase of suicide deaths among young adults [14, 15]. Analyses of risk, protective, and developmental factors leading to a suicide attempt can enhance our understanding of suicidal behaviors. As an example, focusing on the life events of adolescents and young adults, as well as college-age students (i.e., youths in China between 18 and 24 years) can be instrumental in increasing our understanding of factors associated with suicidal behavior. Because individuals at this stage are just entering adulthood, they can offer valuable information that could help prevent further suicidal attempts. The cost to families and society, as well as economic costs are high also for this age group [16]. Thus, evaluating how youth with a history of suicide attempt function psychologically is important to further understand reasons for suicide attempts and for developing suicide prevention strategies.

Therefore, the main purpose of the current study was to examine whether there were any differences between individuals who have attempted suicide and those who have not done so on risk (depression, hopelessness, and psychache) and protective measures (social support, self-esteem, and purpose in life) for suicidality.

## Methods

### Participants

A total of 11,806 students were recruited from seven provinces in China as potential participants. One university was selected through convenience sampling from each of the following provinces: Ningxia, Shandong, Shanghai, Jilin, Qinghai, Xinjiang, and Shaanxi. Potential participants had to be between the ages of 18–22 to be eligible to participate in this study. Participants were excluded if: (1) responses given were deemed implausible i.e. out-of-range, (2) not all items of the SBQ-R were completed, and/ or (3) there was missing key demographic information, such as gender and age.

Of the sample, 237 met criteria for inclusion in a “Suicide Attemper” group. From the remaining 11,569 responses, a simple random sample of 1185 control cases (classified as the “Non-Suicide Attempter” group) was drawn using the random number generators function in the SPSS. A controls to cases ratio of 5:1 was implemented as it is associated with the lowest bias and a higher precision [17, 18]. Responses to an item on the Suicidal Behaviors Questionnaire-Revised [19] were used to select the study participants (see Measures and Procedures subsection).

### Measures and procedures

We used a paper-based survey format to collect data from university students enrolled in undergraduate degree programs between October 2017 and March 2018. Participation was wholly voluntary, with potential study participants provided information about the study. Individuals who agreed to participate provided written informed consent. The questionnaire packets were administered in small group sessions. An equal number of classes from each year of study were then selected to obtain a reasonable representation of each grade.

The data collection included the following self-report instruments:

#### Suicidality

Osman and colleagues [19] developed the Suicidal Behaviors Questionnaire-Revised (SBQ-R) to assess several parameters of suicide-related behaviors, including past attempts, frequency of suicidal ideation, and likelihood of future attempts. Also, they recommended the use of scores on Item 1 (history of attempts) or the SBQ-R total scores for developing subgroups of individuals with history of suicidal attempt(s) and those who have never attempted suicide. The following question from Item 1 “Have you ever thought about or attempted to kill yourself?” was used as the basis to form two groups:

*Suicide Attempter Group*. Participants who endorsed the response option of: *I have attempted to kill myself, but did not want to die* or *I have attempted to kill myself, and really hoped to die* were assigned to the Suicide Attempter group.

*Non-Suicide Attempter Group*. Participants who endorsed the response option of either *never,* or *It was just a brief passing thought* provided was considered the Non-Suicide Attempter group. As noted previously, we used the random extraction procedure to obtain a simple random sample of 1185 control cases.

#### Hopelessness

The Beck Hopelessness Scale (BHS) was used to measure negative attitudes about the future [20] Comprising of 20 items, the response options for this scale were modified from yes/ no to a 5-point Likert-type scale, which ranged from 1 (*strongly agree*) to 5 (*strongly disagree*). The Chinese version of the BHS was found to have acceptable reliability and validity among Chinese university students [21].

#### Depression (DASS-42)

Comprising 42 items, the Depression Anxiety Stress Scales (DASS) was designed to assess negative emotional states according to three subscales, specifically symptoms of depression, anxiety and stress [22–25]. There are 7 items per subscale, rated on a 4-point scale which ranges from 0 to 3, with lower scores on the DASS-42 suggesting better mental health. The instrument has been widely used in China for various psychosocial studies, such as Cheng et al. [26] which reported an internal consistency of Cronbach’s α = 0.77. For this study, we used the depression (DASS-Depression) sub-score.

#### Psychache

The Psychache Scale consists of 13 items reflecting psychache (i.e., mental pain) scored from 1 to 5, with higher scores denoting greater levels of agreement [27]. The Psychache Scale scores has acceptable psychometric properties. Coefficient-α estimates of 0.94 was found when completed by Chinese university students [7].

#### Social support

The Multidimensional Scale of Perceived Social Support (MSPSS) contains 12 items rated on a 7-point Likert-type response scale which measure social support [28]. The total score ranges from 12 to 84, with higher scores on this scale indicating greater social support. MSPSS scale scores have acceptable reliability and validity estimates in Chinese samples [29, 30]. The scale is composed of three sources of social support, including family members, friends, or significant others (or special person). The MSPSS total score is used in this study to reflect overall total support from the three identified sources.

#### Self esteem

The self-esteem scale (SES was originally used to assess adolescents’ overall feelings of self-worth and self-acceptance, and is currently the most widely used self-esteem measure with a good internal reliability estimate (coefficient-α = 0.84) [31, 32]. The SES consists of 10 items with a total score ranging from 10 to 40, where higher scores indicate a greater level of self-esteem. The Chinese SES has been tested in China and found to have acceptable reliability (coefficient-α = 0.71) [33].

#### Purpose in life

The Purpose In Life Test–Short Form comprises four items which measures the extent to which respondents feel their lives have meaning and purpose [34]. The instrument has a 7-point Likert-type scale response format. The total score can range from 4 to 28, with a higher score indicative of greater perceived meaning/ purpose in life. The Chinese version of the PIL-SF has acceptable reliability (coefficient-α = 0.89) [35].

### Statistical analyses

Data normality was assessed using the Shapiro-Wilk test and visual inspection of Q-Q plots. All categorical data was presented using frequency and percentage, while continuous data was summarised using mean ± standard deviation (SD) and median (minimum and maximum) values.

Since all data were not normally distributed, therefore, nonparametric Mann-Whitney *U* test was used to examine whether there were significance differences in the median value of the suicide attempter vs. non-suicide attempter groups on the risk and protective measures.

#### Comparison of risk factors

Scores on three commonly used risk measures for suicidality were adopted to compare the responses of the two groups. The three scores were from the Beck Hopelessness Scale, the Holden Psychache Scale, and the DASS-21 Depression Sub-Scale. A *p*-value of < 0.05 was considered significant for all tests. Effect size using Cohen’s *d* was calculated for each factor.

#### Comparison of protective factors

Three protective factors against suicidality commonly used internationally and also in China were selected and used to compare the two groups (suicide attempters and non-suicide attempters) to measure which group has higher protective factors against suicidality, thus, more unlikely to attempt suicide. The three protective factors selected for the study were Zimet’s MSPSS, Rosenberg’s Self-Esteem Scale, and purpose in life measured by PIL-SF. A *p*-value of < 0.05 was considered significant for all tests. Effect size using Cohen’s *d* for each of the three measurements were also reported.

#### Binary logistic multiple regression analysis

A binary logistic multiple regression was conducted to predict attempt status (suicide attempters versus non-suicide attempters) using the three protective (Social Support, Self Esteem, and Purpose in Life) and three risk factors (Hopelessness, Psychache, and Depression), which was controlled for by age and gender. A *p*-value of < 0.05 was considered significant and odds ratios (OR) were reported with the 95% confidence intervals.

#### Software

All analyses were performed using SPSS version 27 (IBM Corp, Armonk, NY, 2020). A *p*-value of < 0.05 was considered significant for all tests. This study received ethics approval from the institutional review board of the Ethics Committee at the School of Public Health, Shandong University (No. 20161103). Written informed consent was obtained from all participants.

## Results

### Descriptives

Of the 237 suicide attempters, *n* = 96 (40.5%) were male, while the remaining *n* = 141 (59.5%) were female. The mean age for males was 20.70 ± 1.32 and 20.61 ± 1.35 for females. There was no significant difference in mean age between males and females (see Table 1).

**Table 1:** Demographics of Study Participants (*N* = 1422)

Demographics	Suicide Attempt		Total	Statistic	***p***-value	Cohen ***d***
	Yes (*n* = 237)	No (*n* = 1185)	(*n* = 1422)			
Age (years)						
Mean ± SD	21 ± 0.09	21 ± 0.04	21 ± 0.04	1.44	0.150^a^	1.36
Median (min, max)	20 (18, 24)	21 (18, 24)	21 (18, 24)	132,499.00	0.159^b^	0.08^d^
Gender (n, %)						
Male	96 (41%)	444 (38%)	540 (38%)	0.77	0.379^c^	NA
Female	141 (59%)	741 (62%)	882 (62%)			

1. There is no significant difference between mean age for male and female

2. Out of total population sample of 11,806, 237 students reported to have attempted suicide before (based on answer option of Item 1 who responded 4(a) or 4(b)). These 237 participants are grouped under the “Suicide Attempter” group

3. Out of the remaining sample of 11,569 (11,806–237), simple random sampling using SPSS random number generators function is done to extract 1185 samples and these participants are grouped under the “Non-Suicide Attempter” group

4. ^a^Independent samples t-test; ^b^Mann-Whitney U test; ^c^Pearson Chi-Square Test

5. ^d^Calculation obtained from https://www.psychometrica.de/effect_size.html

### Suicidality measurement

The attempters group scored significantly (*p* < 0.001) higher on every measure of suicide risk compared to the non-attempter’s group. The attempters recorded a higher frequency of suicide ideation, higher suicide threats and communication to others of their desire to attempt suicide and self-reported a higher likelihood of attempting suicide in the future. More details on the suicide attempters group score comparison with non-suicide attempters’ group are depicted in Table 2 (See Table 2).

**Table 2:** Comparison between Suicide Attempters and Non-Suicide Attempters Based on Items in the Suicide Behavior Questionnaire-Revised (SBQ-R)

SBQ-R	Suicide Attempt		Total	Statistic	***p***-value	Cohen’s ***d***
	Yes (***n*** = 237)	No (***n*** = 1185)	(***n*** = 1422)			
**Past-Year Ideation Frequency**						
Mean ± SD	1.81 ± 1.05	0.32 ± 0.68	0.57 ± 0.02	20.90	< 0.001^a^	0.75
Median (min, max)	2 (0, 3)	0 (0, 3)	0 (0, 3)	240,548.00	< 0.001^b^	1.04^c^
**Life-Time Threat (Inform Someone)**						
Mean ± SD	0.63 ± 0.48	0.11 ± 0.31	0.19 ± 0.40	16.11	< 0.001^a^	0.34
Median (min, max)	1 (0, 1)	0 (0, 1)	0 (0, 1)	214,248.00	< 0.001^b^	0.72^c^
**Likelihood of Future Attempt**						
Mean ± SD	0.50 ± 0.50	0.05 ± 0.21	0.12 ± 0.33	13.79	< 0.001^a^	0.28
Median (min, max)	1 (0, 1)	0 (0, 1)	0 (0, 1)	204,531.00	< 0.001^b^	0.62^c^

1. Past-Year Ideation Frequency is derived from response to Q2; Life-Time Threat from Q3; and Likelihood of Future Attempt from Q4 of the SBQ-R. Q1 is omitted

2. ^a^Independent samples *t*-test; ^b^Mann-Whitney *U* test

3. ^c^calculation obtained from https://www.psychometrica.de/effect_size.html

Ref: Cohen (1992), .00 to .19 as trivial; values .20 to .49 as small effect, values .50 to .79 as medium effect, and values ≥ .80 as a large effect

Past year ideation frequency. The suicide attempters group had a *Mean* score of 1.81 ± 1.05 and a *Median* score of 2 (*min* = 0, *max* = 3) on past year suicide ideation frequency, while non-suicide attempters had a *Mean* score of 0.32 ± 0.68 and a *Median* score of 0 (0, 3). The difference was significant (*U* = 240,548.00, *p* < 0.001). Effect size using Cohen’s *d* was large (1.04).

Life-Time threat (informing someone). For the measure on ‘informing someone of my intention to kill myself’, the suicide attempters group had a *Mean* score of 0.63 ± 0.48 and a *Median* score of 1 (*min* = 0, *max* = 1), while non-suicide attempters had a *Mean* score of 0.11 ± 0.31 and a *Median* score of 0 (0, 1). The difference was significant (*U* = 214,248.00, *p* < 0.001). Effect size using Cohen’s *d* was medium (0.72).

Likelihood of future attempt. On self-reported likelihood on attempting another suicide, the suicide attempters group had a *Mean* score of 0.50 ± 0.50 and a *Median* score of 1 (*min* = 0, *max* = 1), while non-suicide attempters had a *Mean* score of 0.05 ± 0.21 and a *Median* score of 0 (0, 1). The difference was significant (*U* = 204,531.00, *p* < 0.001). Effect size using Cohen’s *d* was medium (0.62).

### Risk factors for suicide

The attempters group also scored significantly (*p* > 0.001) higher on every measure of risk factor on suicide, i.e. hopelessness, psychache, and depression, compared to non-attempters’ group (See Table 3).

**Table 3:** Comparison between Suicide Attempters and Non-Suicide Attempters Based on Risk and Protective Measures

Factors	Suicide Attempt		Total	Statistic	*p*-value	Cohen’s *d*
	Yes (*n* = 237)	No (*n* = 1185)	(*n* = 1422)			
Risk Measures						
Hopelessness						
• Mean ± SD	55.0 ± 8.4	50.4 ± 9.4	51.1 ± 9.4	7.54	< 0.001^a^	9.21
• Median (min, max)	56 (32, 79)	50 (27, 91)	51 (27, 91)	170,489.00	< 0.001^b^	0.28^c^
Psychache						
• Mean ± SD	33.7 ± 12.2	23.7 ± 9.9	25.4 ± 11.0	11.80	< 0.001^a^	10.31
• Median (min, max)	32 (13, 65)	22 (13, 65)	23 (13, 65)	203,121.00	< 0.001^b^	0.60^c^
Depression						
• Mean ± SD	8.8 ± 5.3	4.7 ± 4.3	5.4 ± 4.7	11.17	< 0.001^a^	4.45
• Median (min, max)	9 (0, 21)	4 (0, 21)	4 (0, 21)	200,905.00	< 0.001^b^	0.58^c^
Protective Measures						
Social Support						
• Mean ± SD	56.7 ± 13.2	62.1 ± 11.8	61.2 ± 12.2	5.83	< 0.001^a^	12.07
• Median (min, max)	57 (15, 84)	62 (12, 84)	61 (12, 84)	101,720.00	< 0.001^b^	0.36^c^
Self Esteem						
• Mean ± SD	25.4 ± 3.9	27.3 ± 3.7	27.0 ± 3.8	6.87	< 0.001^a^	3.70
• Median (min, max)	25 (12, 37)	27 (14, 37)	26 (12, 37)	97,582.00	< 0.001^b^	0.40^c^
Purpose in Life						
• Mean ± SD	18.2 ± 5.1	20.4 ± 4.6	20.0 ± 4.8	6.72	< 0.001^a^	4.69
• Median (min, max)	19 (4, 28)	21 (4, 28)	20 (4, 28)	97,632.50	< 0.001^b^	0.40^c^

1. Suicide Attempters group scored significantly higher (*p* < 0.001) for all three risk factors measurements of hopelessness, psychache, and depression. Size effects using Cohen’s *d* is small for hopelessness, and large for psychache and depression

2. For three protective factors tested, Suicide Attempters group scored significantly lower (*p* < 0.001) for social support, self-esteem and purpose in life. Size effects using Cohen’s *d* is small for social support and self-esteem, while it has medium size effects for purpose in life

3. ^a^Independent samples *t*-test; ^b^Mann-Whitney *U* test

4. ^c^calculation obtained from https://www.psychometrica.de/effect_size.html

Ref: Cohen (1992), .00 to .19 as trivial; values .20 to .49 as small effect, values .50 to .79 as medium effect, and values ≥ .80 as a large effect.

#### Hopelessness

The suicide attempters group had a *Mean* score of 55.0 ± 8.4 and a *Median* score of 56 (*min* = 32, *max* = 79), while non-suicide attempters had a *Mean* score of 50.4 ± 9.4 and a *Median* score of 50 (27, 91). The difference was significant (*U* = 170,489.00, *p* < 0.001). Effect size using Cohen’s *d* was small (0.28).

#### Psychache

For psychache, another risk factor for suicidality, the suicide attempters group had a *Mean* score of 33.7 ± 12.2 and a *Median* score of 32 (*min* = 13, *max* = 65), while non-suicide attempters had a *Mean* score of 23.7 ± 9.9 and a *Median* score of 22 (13, 65). The difference was significant (*U* = 203,121.00, *p* < 0.001). Effect size using Cohen’s *d* was medium (0.60).

#### Depression

The suicide attempters group had a *Mean* score of 8.8 ± 5.3 and a *Median* score of 9 (*min* = 0, *max* = 21), while non-suicide attempters had a *Mean* score of 4.7 ± 4.3 and a *Median* score of 4 (0, 21). The difference was significant (*U* = 200,905.00, *p* < 0.001). Effect size using Cohen’s *d* was medium (0.58).

### Protective factors against suicide

The attempters group scored significantly lower (*p* < 0.001) for all three measures of protective factors comprising social support, self-esteem, and purpose in life.

#### Social support

For total social support score, the suicide attempters group had a *Mean* score of 56.7 ± 13.2 and a *Median* score of 57 (*min* = 15, *max* = 84), while non-suicide attempters had a *Mean* score of 62.1 ± 11.8 and a *Median* score of 62 (12, 84). The difference was significant (*U* = 101,720.00, *p* < 0.001). Effect size using Cohen’s *d* was small (0.36).

#### Self-esteem

For self-esteem, the suicide attempters group had a *Mean* score of 25.4 ± 3.9 and a *Median* score of 25 (*min* = 12, *max* = 37), while non-suicide attempters had a *Mean* score of 27.3 ± 3.7 and a *Median* score of 27 (14, 37). The difference was significant (*U* = 97,582.00, *p* < 0.001). Effect size using Cohen’s *d* was small (0.40).

#### Purpose in life

On measurement for purpose in life, the suicide attempters group had a *Mean* score of 18.2 ± 5.1 and a *Median* score of 19 (*min* = 4, *max* = 28), while non-suicide attempters had a *Mean* score of 20.4 ± 4.6 and a *Median* score of 21 (4, 28). The difference was significant (*U* = 97,632.50, *p* < 0.001). Effect size using Cohen’s *d* was small (0.40).

#### Suicide attempt risk

The binary logistic regression model on factors predicting suicide attempt was significant, χ^2^(8) = 224.65, *p* < 0.001, explaining 11.4% (Nagelkerke R^2^) of variance in suicide attempt and had classified correctly 98% of cases. The Hosmer and Lemeshow test requires that *p* > 0.05 for a good fit. In the present analysis, *p* = 0.252, and so the model was a good fit for the data. The regression results indicated that those who were more depressed had the highest risk of suicide attempts (OR 1.123, 95% CI: 1.076, 1.173, *p* < 0.001); they also had a high psychache score (OR 1.045, 95% CI: 1.028, 1.063, *p* < 0.001). Younger age was most protective against suicide attempts (OR 0.870, 95% CI: 0.783, 0.967, *p* < 0.001) (Refer to Table 4).

**Table 4:** Binary logistic regression of factors predicting suicide attempt

Variable	Wald	OR	95% CI		***p***-value
			Lower	Upper	
Constant	0.115				
Hopelessness	6.268	0.973	0.952	0.994	0.012
Psychache	27.017	1.045	1.028	1.063	< 0.001
Depression	28.357	1.123	1.076	1.173	< 0.001
Social Support	7.061	0.984	0.973	0.996	0.008
Self-Esteem	0.000	1.000	0.953	1.048	0.990
Purpose In Life	0.711	0.987	0.958	1.017	0.399
Age	6.684	0.870	0.783	0.967	0.010
Gender					
Male^a^					
Female	0.960	1.152	0.868	1.527	0.327

*OR* Odds Ratio, *CI* Confidence Interval. ^a^Reference Group

## Discussion

Our findings indicate that suicide attempters had higher past-year ideation, life-time threat, and likelihood of future attempt compared to their counterparts who had made no previous suicide attempt. Suicide attempters also indicated higher risk factors, namely greater hopelessness, psychache, and depression, and significantly lower protective factors (social support, self-esteem, and purpose in life) in the univariate analyses. However, in the multiple regression model, only psychache and depression remained significant risk factors, and social support and younger age were protective factors. Hopelessness had become a significant protective factor, but the results may be spurious as the effect sizes in the *t*-test and multivariate analyses were small and negligible.

The results on psychache, depression, and social support as predictors of suicide attempt is consistent with a number of past studies carried out within and outside of China [6–11]. The results suggest that the suicide attempter group had higher risks of suicidality compared to the non-attempter group.

From the research, it seems that the suicide attempters still reported psychological characteristics akin to a potential suicide attempter’s, with the high risk of suicidality and high scores on risk factors and low scores on protective factors. The high suicidality risk may increase the risk of attempting suicide again [5]. Therefore, there is a need to formulate intervention strategies and programmes that draw from an established and agreed upon concept of suicide recovery/rehabilitation for attempters. This should ideally include an understanding of what “recovery” from suicidality means for each individual [36]. Key protective factors should be identified for each individual and worked on by mental health professionals for them to be protective [37]. Such individuals may benefit considerably from psychotherapy or medication. Specifically, cognitive behavioral therapy with the goals of ameliorating depression and a negative view of the self, while building hope and a purpose in life may relieve suicidal behaviors [38]. In addition, universities could implement universal suicide prevention efforts such as gatekeeper training which may indirectly build social support for college students who are struggling with suicidal thoughts [39].

The cross-sectional research design does not allow us to infer causality. Generalization to other universities, provinces, rural areas, other age-groups, or other sections of society cannot be made without further research, given the vastness, diversity, and huge number of populations of a country like China. Context and time lapse from previous suicide attempts, participation in any rehabilitation programmes, whether the participants attempted suicide only once or multiple times are not known.

Future research can focus on ways and means to reduce risk factors and increasing protective factors for surviving suicide attempters, especially in following up with their case after discharging from hospital care. Universities can also include this important suicide prevention risk management into its programmes. There is a need to further explore attitudes towards suicide among adolescents, with further attempts at diversity and inclusion [40, 41]. Increasing awareness of and training healthcare professionals to better identify suicide may be worth exploration as future avenue for further research in this area [42–44]. A proper rehabilitation and monitoring programme may be necessary with the longer-term objective of reducing the risk factors and to nurture the protective factors.

## Conclusions

This study indicates that undergradate students who reported a previous suicide attempt continued to have higher scores of risk factors and lower scores of protective factors. The results draws concern that these at-risk students may continue to be at a higher likelihood of a suicide attempt. Therefore, the appropriate clinical interventions shoud be identified to reduce their risk of reattempting, and to improve their psychological well-being in general.

## Data Availability

The datasets used and/or analysed during the current study are available from the corresponding author on reasonable request.

## Abbreviation

SBQ-R: Suicidal Behaviors Questionnaire-Revised

## References

[1] World Health Organization (2017). Depression and other common mental disorders: global health estimates.

[2] World Health Organization (2019). Global Health Observatory data repository: suicide rates estimates, crude.

[3] Cerel J, Brown MM, Maple M, Singleton M, van de Venne J, Moore M (2019). How many people are exposed to suicide? Not six. Suicide Life Threat Behav.

[4] Horwitz AG, Czyz EK, King CA (2015). Predicting future suicide attempts among adolescent and emerging adult psychiatric emergency patients. J Clin Child Adolesc Psychol.

[5] Beautrais AL (2004). Further suicidal behavior among medically serious suicide attempters. Suicide Life Threat Behav.

[6] Liu Y, Zhang J, Sun L (2017). Who are likely to attempt suicide again? A comparative study between the first and multiple timers. Compr Psychiatry.

[7] Lew B, HuenJ, Yu P, YuanL, WangD-F,Ping F, et al. Associations between depression, anxiety, stress, hopelessness, subjective well-being, coping styles and suicide in Chinese university students. PLoS ONE. 2019;14(7):e0217372. 10.1371/journal.pone.0217372. PMC6602174 31260454

[8] Ribeiro JD, Huang X, Fox KR, Franklin JC (2018). Depression and hopelessness as risk factors for suicide ideation, attempts and death: meta-analysis of longitudinal studies. Br J Psychiatry.

[9] Troister T, D'Agata MT, Holden RR (2015). Suicide risk screening: comparing the Beck depression inventory-II, Beck hopelessness scale, and Psychache scale in undergraduates. Psychol Assess.

[10] Soto-Sanz V, Piqueras JA, Perez-Vazquez MT, Rodriguez-Jimenez T, Castellvi P, Miranda-Mendizabal A, Pares-Badell O, Almenara Barrios J, Blasco MJ, Cebria A, Gabilondo A (2019). Self-esteem and suicidal behaviour in youth: a meta-analysis of longitudinal studies. Psicothema..

[11] Miller AB, Esposito-Smythers C, Leichtweis RN (2015). Role of social support in adolescent suicidal ideation and suicide attempts. J Adolesc Health.

[12] Rodríguez SP, Salvador JH, García-Alandete J (2017). The role of hopelessness and meaning in life in a clinical sample with non-suicidal self-injury and suicide attempts. Psicothema..

[13] Wang Z, Wang J, Bao J, Gao X, Yu C, Xiang H (2016). Temporal trends of suicide mortality in mainland China: results from the age-period-cohort framework. Int J Environ Res Public Health.

[14] Fei F, Liu H, Leuba SI, Li Y, Hu R, Yu M, et al. Suicide rates in Zhejiang Province, China, from 2006 to 2016: a population-based study. J Epidemiol Community Health. 2019;73:745-9. 10.1136/jech-2018-211556 PMC6678047 30992370

[15] Zhao S, Zhang J (2015). Suicide risks among adolescents and young adults in rural China. Int J Environ Res Public Health.

[16] Kinchin I, Doran C (2018). The cost of youth suicide in Australia. Int J Environ Res Public Health.

[17] Linden A, Samuels SJ (2013). Using balance statistics to determine the optimal number of controls in matching studies. J Eval Clin Pract.

[18] Ury HK. Efficiency of case-control studies with multiple controls per case: continuous or dichotomous data. Biometrics. 1975:643–9. 1100136

[19] Osman A, Bagge CL, Gutierrez PM, Konick LC, Kopper BA, Barrios FX (2001). The suicidal behaviors questionnaire-revised (SBQ-R): validation with clinical and nonclinical samples. Assessment..

[20] Beck AT, Weissman A, Lester D, Trexler L (1974). The measurement of pessimism: the hopelessness scale. J Consult Clin Psychol.

[21] Yuan L, Wang DF, Lew B, Osman A, Jia CX (2018). The future disposition Inventory-24: reliability and validity estimates in a large sample of Chinese University students. BMC Psychiatry.

[22] Lovibond PF, Lovibond SH (1995). The structure of negative emotional states: comparison of the depression anxiety stress scales (DASS) with the Beck depression and anxiety inventories. Behav Res Ther.

[23] Henry JD, Crawford JR (2011). The short-form version of the depression anxiety stress scales (DASS-21): construct validity and normative data in a large non-clinical sample. Br J Clin Psychol.

[24] Osman A, Wong JL, Bagge CL, Freedenthal S, Gutierrez PM, Lozano G (2012). The depression anxiety stress Scales-21 (DASS-21): further examination of dimensions, scale reliability, and correlates. J Clin Psychol.

[25] Szabo’ M (2010). The short version of the depression anxiety stress scales (DASS-21): factor structure in a young adolescent sample. J Adolesc.

[26] Cheng Q, Kwok C, Zhu T, Guan L, Yip P (2015). Suicide communication on social media and its psychological mechanisms: an examination of Chinese microblog users. Int J Environ Res Public Health.

[27] Holden RR, Mehta K, Cunningham EJ, McLeod LD (2001). Development and preliminary validation of a scale of psychache. Can J Behav Sci.

[28] Zimet GD, Dahlem NW, Zimet SG, Farley GK (1988). The multidimensional scale of perceived social support. J Pers Assess.

[29] Wang Y, Wan Q, Huang Z, Huang L, Kong F (2017). Psychometric properties of multi-dimensional scale of perceived social support in Chinese parents of children with cerebral palsy. Front Psychol.

[30] Chou KL (2000). Assessing Chinese adolescents’ social support: the multidimensional scale of perceived social support. Personal Individ Differ.

[31] Lakey CE, Hirsch JK, Nelson LA, Nsamenang SA (2014). Effects of contingent self-esteem on depressive symptoms and suicidal behavior. Death Stud.

[32] Rosenberg M (1965). Society and the adolescent self-image.

[33] Zhang J, Norvilitis JM (2002). Measuring Chinese psychological well-being with Western developed instruments. J Pers Assess.

[34] Schulenberg SE, Schnetzer LW, Buchanan EM (2011). The purpose in life test-short form: development and psychometric support. J Happiness Stud.

[35] Law BM. Psychometric properties of the existence subscale of the purpose in life questionnaire for Chinese adolescents in Hong Kong. Sci World J. 2012;2012. 10.1100/2012/685741 PMC3425806 22927785

[36] Sellin L, Asp M, Wallsten T, Wiklund GL (2017). Reconnecting with oneself while struggling between life and death: the phenomenon of recovery as experienced by persons at risk of suicide. Int J Ment Health Nurs.

[37] Conway C, Surgenor PW, Thekiso TB, Moore A, Campion A, Tormey A (2018). Client personal recovery and recovery orientation of an Irish suicide intervention charity. Ir J Psychol Med.

[38] Li W, Dorstyn DS, Jarmon E (2020). Identifying suicide risk among college students: a systematic review. Death Stud.

[39] Wolitzky-Taylor K, LeBeau RT, Perez M, Gong-Guy E, Fong T (2020). Suicide prevention on college campuses: what works and what are the existing gaps? A systematic review and meta-analysis. J Am Coll Heal.

[40] Aen MA, Ibrahim N, Che DN (2017). A narrative review of the relationship between victimization, depression and suicide ideation among lesbian, gay and bisexual individuals. Int J Indian Psychol.

[41] Siau CS, Wee LH, Yacob S, Yeoh SH, Binti Adnan TH, Haniff J, Perialathan K, Mahdi A, Rahman AB, Eu CL, Binti WS (2017). The attitude of psychiatric and non-psychiatric health-care workers toward suicide in Malaysian hospitals and its implications for training. Acad Psychiatry.

[42] Siau CS, Wee LH, Ibrahim N, Visvalingam U, Yeap LL, Wahab S (2018). Gatekeeper suicide training's effectiveness among Malaysian hospital health professionals: a control group study with a three-month follow-up. J Cont Educ Health Prof.

[43] Ibrahim N, Din NC, Amit N, Ghazali SE, Safien AM. Development and validation of Yatt Suicide Attitude Scale (YSAS) in Malaysia. PLoS One. 2019;14(2). 10.1371/journal.pone.0209971 PMC6392240 30811425

[44] Siau CS, Wee LH, Ibrahim N, Visvalingam U, Wahab S (2017). Cross-cultural adaptation and validation of the attitudes toward suicide questionnaire among healthcare personnel in Malaysia. Inquiry..

